# Suicide and depressive symptoms possible correlates among a sample of Egyptian physicians: observational cross-sectional study (online survey)

**DOI:** 10.1186/s12888-024-05825-w

**Published:** 2024-05-30

**Authors:** Mohamed A. Khalil, Dalia Khalifa, Rasha Mahmoud Allam, Shaimaa Abdalaleem Abdalgeleel, Ola Osama Khalaf

**Affiliations:** 1 Psychiatry Department, Faculty of Medicine, Cairo University, Cairo, Egypt; 2 Cancer Epidemiology and Biostatistics Department, National Cancer Institute, Cairo University, Egypt; 3 Department of Public Health, Faculty of Applied Medical Sciences, Al-Baha University, Al-Baha, Saudi Arabia

**Keywords:** Depression, Egyptian physicians, Suicidal ideation

## Abstract

**Background:**

Compared to other occupations, physicians are more susceptible to depression and suicide. Suicide among physicians in some countries reached up to 1.5- to threefold higher than the general population. However, this rate was not homogenous in all countries. Most of the Egyptian studies were related to the stressful pandemic event, but the actual prevalence of depression among physicians is still under research. To the best of the researcher's knowledge, no other study has been conducted to evaluate the risk of suicide among Egyptian physicians.

**Aim:**

The study aimed to screen for depressive symptoms and suicide among Egyptian physicians and to investigate the correlates associated with suicide ideations.

**Methods:**

This cross-sectional survey included Egyptian physicians recruited online by Google Forms. Depressive symptoms were screened using the Beck Depression Scale (BDI-II), while suicidal ideas were assessed using the Suicidal Ideation Attributes Scale (SIDAS).

**Results:**

Six hundred sixty Egyptian physicians completed the survey following a two-week pilot study between January 10 and July 16, 2023. The average age was 39.1 years, and 71.4% were married. 49.1% were medical specialists. The median daily working hours were eight, and 27.7% of the physicians attended night shifts. 22.3% had a psychiatric illness, and 34.3% had a chronic disease. Younger and single physicians of both sexes were more prone to suicide risk (*p*-value = 0.019 and 0.021, respectively). Those with psychiatric or chronic medical disorders had a higher suicidal risk (*p*-values < 0.001 and 0.004, respectively). Physicians with fewer academic degrees and those who work longer hours or night shifts had more depressive symptoms (*p*-values < 0.001 and 0.009, respectively). The risk of depression and suicide is almost the same in all medical specialties. The SIDAS suicide score and the Beck depression score revealed a statistically significant association (r = 0.288, *p*-value < 0.001).

**Conclusion:**

Suicide risk is higher among younger, single physicians of both sexes, as well as those with psychiatric or chronic medical disorders. More depressive symptoms are seen in physicians who have more extended hours or night shifts and who have fewer academic degrees. Almost all medical specialties carry the same risk of depression and suicide. Longitudinal research is recommended for regular follow-up of suicidal thoughts and depressive symptoms.

## Introduction

Some occupations have been linked to an increased risk of suicide, including different health professions [1, 2]. Physicians, in particular, have been the subject of various studies on suicide prevalence [3, 4]. Some medical specialties have displayed a higher risk of suicide than others [5, 6]. The suicide rate among physicians has reached up to 1.5–3 times higher than that of the general population [7]. However, this rate was not always homogenous in different countries [8]. Some studies have demonstrated increased suicide rates for both male and female physicians [9, 10]. Others revealed that female physicians had higher suicide rates [11, 12]. This could be explained by the fact that their social family position adds to the workload and creates tension between their many social and professional obligations [13]. Few studies demonstrated that neither gender was at higher risk of suicide [14, 15]. This risk raised alarming concerns at different medical institutional and community levels and revealed the vital need to implement efficient preventive measures [16].

The risk factors for physician suicide are not well studied. For instance, one crucial aspect that differs across nations is the working conditions for doctors. These factors include underpayment, serving too many patients, long duty hours with consequent disruption of home life, and conflict of responsibilities [17, 18]. Furthermore, the comorbidity and prevalence of different psychiatric disorders among physicians, such as major depression and anxiety disorders, play an important role. Studies have demonstrated that residency training, for example, is associated with a high prevalence of severe depression [19] and that more than one-quarter of physicians reported depressive and anxiety symptoms [20]. Anhedonia, in particular, and work dissatisfaction are linked with an increased risk of suicide in physicians [21]. In addition, the stigma related to depression could further prevent adequate seeking of professional psychiatric advice. This, in turn, increases the risk of suicide-related behaviors among physicians, such as searching for suicide-related information, making plans, discussing suicide-related problems with each other, and trying to improve their accessibility to poisonous substances [22, 23].

Other factors that heighten the risk of suicide among physicians are perceived medical errors, increased exhaustion, and high levels of job dissatisfaction [24, 25]. Medical adverse occurrences and mistakes, with their subsequent guilt feelings and frustration, are proven to be substantially correlated with suicide ideations and attempts.

Healthcare workers often seek and expect perfection from themselves, refusing to accept errors or even possible complications. A significant contributing element to the doctor's suicide risk factor is his emotional weight of anxiety, guilt, and sometimes shame after a traumatic, unfavorable medical mistake. It may lead to the emergence of the condition known as "second victim syndrome," in which the physician himself represents the second victim [26–28].

In Egypt, several studies have been conducted in the past few years to investigate the prevalence of depression among physicians. At least about one-fifth of Egyptian physicians displayed depressive symptoms, and other studies even illustrated higher results [29–31]. However, most of these studies were related to the stressful pandemic event, but the actual prevalence of depression among physicians other than this exceptional factor is still under-researched. As far as the researchers know, no studies were performed to assess suicidal risk among Egyptian healthcare professionals. So, this study aimed to screen for depressive symptoms and suicide among Egyptian physicians and to investigate the different correlates with suicidal ideations considering age, gender, working hours, and medical and surgical specialties.

### Subjects and methods

This descriptive study included 660 Egyptian physicians recruited by an online Google Form survey. An invitation to participate in the survey was sent by e-mail and social media to Egyptian physicians working in different governmental and private medical institutes in Cairo. Seven hundred and five physicians responded to the survey, but 45 were excluded due to incomplete data.

In this study, physicians were defined as medical doctors who were awarded a Bachelor of Medicine or a Bachelor of Surgery and had a license from the Egyptian Medical Syndicate to practice medicine in Egypt.

Physicians from all specialties, genders, and ages were included.

Both governmental and private-sector physicians from Cairo were asked to participate. There were no exclusions for any medical or psychiatric morbidities. Data were collected from January 10, 2023, until July 16, 2023. The pilot lasted for two weeks.

In Egypt, the main categories of physician classification are surgical, medical, assistive, and academic [32]. Surgery includes general surgery, obstetrics and gynecology, oncology surgery, anesthesia, orthopedics, and ENT; medical includes general medicine, medical oncology, pediatrics, chest, family medicine, ICU, dermatology, psychiatry, endocrine, and rheumatology; and academic includes public health, physiology, anatomy, histology, pharmacology, pathology, microbiology, and microbiology.

The survey included demographic data, occupational data, an assessment of depressive symptoms using the Beck Depression Scale (BDI-II), and suicidal ideas using the Suicidal Ideation Attributes Scale (SIDAS). 

The Suicidal Ideation Attributes Scale (SIDAS) is a short, valid, self-rated, and web-based scale developed by van Spijker et al. [33] to assess the frequency and controllability of suicidal ideation, closeness to attempt suicide, level of distress associated with the suicidal thoughts, and the impact on daily functioning. The scale consists of five questions that assess suicidal thoughts during the last month. The subject rates every question from zero to ten on a scale, where 0 = no thoughts and 10 = extreme thoughts. Total SIDAS scores are calculated as the sum of the five items, with controllability reversed (10 = 0, 9 = 1, …, 0 = 10). Total scores range from 0 to 50. According to Van Spijker et al. [33], the SIDAS score is classified into no ideation (SIDAS score 0), low ideation (score 1–20), and high ideation (score 21–50). 

The Beck Depression Scale (BDI-II), developed by Beck et al. [34], was used to evaluate depressive symptoms in the participants. It is a 21-item, self-rated scale that assesses critical symptoms of depression. Each item is scored on a 4-point continuum (0 = least, 3 = most), with a total score range of 0 to 63 [35]. The higher the score, the more severe the depression.

The following scoring has been suggested to interpret the BDI-II: minimal range 0–13, mild depression 14–19, moderate depression 20–28, and severe depression 29–63 [19]**.** Becket al. [36] demonstrated the convergent and discriminant validity of the BDI-II in 87 psychiatric outpatients by correlating BDI-II scores with scores from the Hamilton Psychiatric Rating Scale for Depression and the Hamilton Rating Scale for Anxiety.

A pilot study was conducted before the beginning of the actual data collection. The Beck Scale for Suicidal Ideation (BSSI) was used [37]. However, it was reported by three participants that reading the questionnaire had increased their suicidal ideation. In the absence of direct contact with participants, the researchers were worried about increasing suicide risk among some participants. This is why the Suicidal Ideation Attributes Scale (SIDAS) was used instead of the Beck Scale for Suicidal Ideation. No similar comments were received from the participants after using the SIDAS.

### Sample size

The sample size was calculated using the total number of working Egyptian physicians (82,000), according to the Egyptian Medical Syndicate [38], and a previous study discovered that the prevalence of suicidal ideas among Egyptian medical residents was about 25% [39]. At a confidence interval of 99%, a minimal sample of 500 subjects was calculated using Epi Info ™ software (version 7.2.5.0).

### Statistical analysis

The results were analyzed using Statistical Package for the Social Sciences (SPSS) version 27. Numbers and percentages were used to represent qualitative data. The mean and standard deviation (SD) or median (range) of numerical variables were used as applicable. The Kolmogorov–Smirnov single-sample test was used to determine the data's normality. The Mann–Whitney U test was used to compare two independent groups, and the Kruskal–Wallis test was used to compare more than two independent groups, followed by a post hoc test with *p*-value correction using Benferroni correction. Spearman's rho correlation was used to assess the correlation between the Beck and SIDAS scores. A *p*-value equal to or less than 0.05 was considered significant. All tests were two-tailed.

## Results

The study included 660 Egyptian physicians, and the median age of the participants was 39 years, ranging from 23 to 73 years. About three-fourths of the participants were married (71.4%), and about half were medical specialists (49.1%). The median years after graduation were 14 years, ranging from 1 to 50 years; the median working hours per day were eight hours, ranging from 2 to 20; and about one-fourth of the physicians (27.7%) attended the night shift. Nearly one-third of the participants (34.3%) complained of chronic disease, and 22.3% suffered from a psychiatric illness (Table 1).

**Table 1:** Characteristics of the participants

Characteristics	*n* = 660
Age (years)	39 (23–73)^a^
Gender	
Male	303 (45.9%)
Female	357 (54.1%)
Marital status	
Single	141 (21.4%)
Married	471 (71.4%)
Divorced	35 (5.3%)
Widow	13 (2%)
Degree	
Bachelor	85 (12.9%)
Diploma	31 (4.7%)
Master	215 (32.6%)
Egyptian Board	67 (10.2%)
M.D	262 (39.7%)
Physician specialty	
Assistive	67 (10.2%)
Academic	123 (18.6%)
Medical	324 (49.1%)
Surgical	146 (22.1%)
Years after graduation	14 (1–50)^a^
Working hours/day	8 (2–20)^a^
Night shift	
Yes	227 (34.3%)
Previous psychiatric illness presence	
Yes	147 (22.3%)
Chronic illness presence	
Yes	183 (27.7%)

^a^Median (range)

The average depression score was 17.9 ± 10.1. Mild depression was detected in 23.8% (157) of the participants; 28.9% (191) reported moderate depression, 20.3% (134) had severe depression, and 5.3% (35) had more severe symptoms. The average suicide score was 10.9 ± 6.7; low suicidal ideation was discovered in 82.9%, and high suicidal ideation was detected in 7.3% (Table 2).

**Table 2:** Prevalence of depressive symptoms and suicide among physicians

Depression Score (BDI-II)	17.9 ± 10.1^a^
No	143 (21.7%)
Mild	157 (23.8%)
Moderate	191 (28.9%)
Severe	134 (20.3%)
More severe	35 (5.3%)
Suicide Score (SIDAS)	10.9 ± 6.7^a^
No ideation	65 (9.8%)
Low ideation	547 (82.9%)
High ideation	48 (7.3%)

^a^Mean ± standard deviation

Regarding the Beck score for depression, the only statistically significant associated variables were age (*p*-value < 0.001), academic degree (*p*-value < 0.001), working hours/day (*p*-value = 0.009), years after graduation (*p*-value = 0.008), night shift working (*p*-value = 0.001), previous psychiatric illness presence (*p*-value < 0.001), and finally chronic illness presence (*p*-value = 0.004). As regards the SIDAS score for suicide, the only statistically significant associated variables were age (*p*-value = 0.019), marital status (*p*-value = 0.021), previous psychiatric illness presence (*p*-value < 0.001), and finally, chronic illness presence (*p*-value < 0.001) (Table 3). There was a statistically significant correlation; however, it was weak between the Beck score and the SIDAS score (Spearman's rho = 0.288, *p*-value < 0.001) (Table 4).

**Table 3:** Relation of BDI-II, SIDAS score, and sociodemographic characteristics

	BDI-II scores	*p*-value	SIDAS score	*p*-value
Age (years)				
< 39 years	19.4 ± 10.2	< 0.001	11.8 ± 7.9	0.019
≤ 39 years	16.5 ± 9.7		10.1 ± 5.1	
Sex				
Male	17.7 ± 8.4	0.731	10.1 ± 4.8	0.061
Female	18.09 ± 11.2		11.51 ± 7.8	
Marital status				
Married	17.6 ± 9.6	0.351	10.6 ± 6.6	0.021
Not-married	18.7 ± 10.9		11.8 ± 6.9	
Academic degree				
Bachelor^a^	21.3 ± 11.3	< 0.001	11.51 ± 8.5	0.669
Diploma or Master^a^	18.9 ± 9.6		11.02 ± 8.6	
M.D. or Board^b^	16.3 ± 9.7		10.7 ± 6.2	
Specialty				
Assistive	15.7 ± 9.6	0.186	11.5 ± 6.7	0.802
Academic	18.5 ± 10.8		11.1 ± 7.3	
Medical	18.4 ± 10.3		10.8 ± 6.5	
Surgical	17.2 ± 8.6		10.9 ± 6.6	
Working hours/day				
< 8 h	16.8 ± 9.6	0.009	11.1 ± 6.7	0.252
≥ 8 h	18.7 ± 10.1		10.8 ± 6.7	
Years after graduation				
< 14 years	18.94 ± 10.1	0.008	11.5 ± 7.8	0.482
≥ 14 years	17.0 ± 9.9		10.5 ± 5.6	
Night shift				
No	17.2 ± 10.1	0.001	10.8 ± 6.4	0.600
Yes	19.8 ± 9.8		11.3 ± 7.4	
Previous psychiatric illness presence				
No	16.1 ± 8.8	< 0.001	10.4 ± 5.6	< 0.001
Yes	24.2 ± 11.1		14.1 ± 8.7	
Chronic illness presence				
No	16.6 ± 9.2	< 0.001	10.5 ± 6.7	0.004
Yes	20.4 ± 10.9		11.7 ± 6.8	

Cells that shared the same letters are not statistically significant

**Table 4:** Correlation between BDI-II and SIDAS score

	**SIDAS score**	
	**Correlation coefficient (r)**	***p*-value**
**BDI-II score**	0.288	< 0.001

## Discussion

This study aimed at screening for depressive symptoms and suicidal ideations in Egyptian physicians working in Egypt. As far as the researchers know, this is the first study to screen for suicidal ideas in a representative number of Egyptian physicians. Suicidal ideation is the first step in the lengthy process of suicide, so it is considered a specific indicator of suicidal attempts. The link between suicidal ideation and the possibility of suicidal attempts, especially in physicians, is not well studied and is an underestimated phenomenon among physicians in Arabic countries, including Egypt [40]. Medicine is one of the most common professions associated with depression and suicide compared to other jobs or the general population [11, 41].

Interestingly, among the 660 physicians, only 21.7% did not display depressive symptoms, while 25.6% had severe depression. A meta-analysis of 54 studies involving 17,560 physicians detected that the prevalence of depression or depressive symptoms was 28.8%, ranging from 20.9% to 43.2% [42].

Physicians are less likely to receive mental health treatment due to many causes, including lack of time, lack of confidentiality, stigma, and fear of documentation on academic records [40, 42–45]. Thus, the prognosis of depression in physicians may be worsened, leading to an increased risk of suicide. The association between depression and suicidal ideation is more significant and direct than the association between exhaustion and suicidal ideation in physicians [46]. This is why the researchers were more inclined to measure depression than fatigue in the current study. 

The association between depression and suicidal ideas was relatively weak. However, it was significant, probably due to the religious factor that may decrease suicide and the fact that the sample is a community sample, not a sample of patients seeking treatment.

It was discovered that 7.3% of the physicians had a high risk of suicidal behavior, and 82.9% had low to high suicidal ideation. Medical students and physicians have a higher prevalence of suicide than the general population [11, 44, 45]. A systematic review that included studies about medical students worldwide revealed that 27.2% have depressive symptoms and 11.1% have suicidal ideation [47]. A Norwegian survey of 1,000 physicians detected that one-fourth of the physicians had suicidal ideas at least once during their lives; meanwhile, the study by Hem et al. [48] reported that the rate of suicidal attempts was 1.6%; the explanation for this could be due to causes related to the medical profession in general and causes related to Egyptian physicians in particular. Risk factors for suicide in physicians worldwide may include depression, substance abuse, occupation-specific factors, including practicing an increased workload volume, being evaluated as unfit to practice, perceived medical errors, workplace harassment, access to lethal means, and the knowledge to use such means effectively. Occupational settings were described as particularly stressful, such as working in emergency departments with a high prevalence of shift work, exposure to aggressive and violent behavior from patients and situations relating to trauma, a lack of appropriate training, and financial motivations, in addition to the possible effects of the COVID-19 epidemic due to the long periods of lockdown and working in isolation hospitals [20, 49–52].

Moreover, the Egyptian community has been suffering from an increase in suicide in the last few years due to multiple factors like financial stressors, increasing social conflicts, substance use, violence, and the post-COVID-19 effects. Furthermore, psychiatric services were deficient in addition to the stigma about suicide in the community [53, 54]. Physicians in Egypt have specific risk factors for suicide as health systems are poorly developed, resources are deficient, and the increasing number of immigrants among Egyptian physicians led to a decreased number of working physicians in Egypt, according to the Egyptian Medical Syndicate, putting more workloads on them [20, 38]. However, strong social support and religious beliefs—as more than 99% of the population are either Muslims or Orthodox Christians—help in decreasing the suicide rates in comparison to the worldwide average. According to WHO statistics, Egypt has a suicide rate of 3.4 per 100,000 people, compared to an average of 9.0 worldwide [53, 55]. This low rate of suicide may additionally be due to defects in surveillance leading to under-reporting consequences.

In the present study, younger age was significantly associated with more depression and suicidal ideation; this is in line with research by Ventriglio et al. [56], which reported a higher prevalence of psychiatric disorders among younger physicians. This may be due to a lack of experience coping with occupational stressors that add to their unsettled financial position and extra academic stressors as they usually join postgraduate studies. In the same direction, physicians with fewer years of experience and lower academic grades had significantly higher levels of depression. These findings are similar to other studies that reported higher suicidal ideas in physicians below 45 years old [57, 58]. Meanwhile, a large study in Australia conducted by Petrie et al. [59] did not report age differences.

Notably, females scored higher in depression and suicidal ideation than males, although these differences were not statistically significant. Most studies have discovered increased depression and suicidality in females among the general population and physicians [11, 48, 59, 60]. Studies focusing on suicidal thoughts rather than behavior revealed inconsistent findings. Van Spijker et al. [33] reported no significant differences between males and females regarding suicidal ideation. Similarly, an Egyptian study demonstrated no gender difference in depression among physicians [38]. Some studies have proven that males have even more suicidal thoughts than females, with no difference in depression between the two genders [56–59, 61–63]. The male-to-female ratio of suicide is higher in physicians than in the general population [63]. The WHO report about rates of suicide in Egypt stratified by gender reported higher rates in males than females [55]. The more stressful work environments men face in surgical specialties and isolation hospitals during epidemics could contribute to these findings.

Furthermore, men in Egypt face a more significant financial burden due to social conventions than women do. Substance abuse is much more prevalent in men and is a considerable risk factor for suicidality among physicians. Lastly, in a conservative society like Egypt, men may feel less shame compared to women when discussing thoughts of suicide. Consequently, males may surpass females in reporting suicide attempts rather than completed suicides.

However, there are additional risk factors for suicide ideation that are specific to women, including hormonal fluctuations, a higher frequency of depression, stressors associated with the childbearing period, such as menstruation, pregnancy, and lactation, sexual abuse, and discrimination based on gender [42].

Married physicians had significantly less suicidal ideation than single physicians, a result that replicated previous studies [20, 64]. A different result was reported by Gold et al. [63], who reported increased deaths due to suicide among married physicians. A study by Mohamed et al. [39] did not find differences in suicidal ideation among Egyptian physicians with different marital statuses; however, most of the included physicians were young and single.

In this study, increased working hours and night shifts were significantly associated with more depression in physicians but not with suicidal ideas. However, other studies reported that increased psychiatric disorders and suicidal ideation were associated with increasing working hours for physicians [40, 64, 65]. The wrong reporting or the smaller sample size may explain the difference in the present study.

The researchers did not detect a significant difference between medical specialties in either depression or suicidal ideation severity. Contradictory results have been reported in the literature about the specialties that have the highest risks of suicide. Many studies have reported that psychiatry, anesthesia, and surgery have the highest rates of suicide among their practitioners [5, 56, 59, 66]. In comparison, an Egyptian study by Mohamed et al. [39] discovered the highest rate of depression in pediatric physicians and suicidal thoughts in obstetrics and gynecology physicians. A meta-analysis including the results of eight studies that discussed the effect of medical specialties on physician suicide concluded that general practitioners, followed by internal medicine practitioners, are at the highest risk for suicide among all specialties [4]. Another meta-analysis of 54 studies reported no difference between surgical and non-surgical resident physicians [19]. The social differences between the studied communities and the differences in the work systems and roles of each specialty can explain the inconsistency of these results.

Participants with psychiatric disorders were discovered to have a higher level of suicidal ideas, a finding that replicates the results of previous studies [5, 11, 20, 25]. However, psychiatric disorders were not diagnosed using a structured interview, which decreased the reliability of such results. Depression and suicidal ideation were higher in physicians with chronic medical illnesses. Similar findings were reported by Ji et al. [66], who reported that having a medical disease increased the odds of suicide by one and a half times.

### Limitations

The online access to the physicians in this study can be considered a strength and a limitation. In Egypt, like most Arabian and Muslim countries, suicide is regarded as a stigma. Many Egyptians may hesitate to discuss suicidal thoughts with others. The anonymity of the online survey may have helped the participants express their thoughts more honestly. However, the subjective nature of the study and the absence of objective clinical judgment of the participant's symptoms may decrease the credibility of the collected data and increase the possibility of under- or over-reporting of suicide and depression. Moreover, since it was an online survey and choosing to participate was optional, not all specialties were equally presented. More social and financial data should be collected in further studies and correlated with the clinical findings.

Further assessments of other suicide risk factors like weariness and substance use, for example, would add more value to the results of the present research. Follow-up of both depressive symptoms and suicidal ideation at regular intervals may add more value to the study, and longitudinal research, including a case–control design, is recommended. More studies, including direct physician interviews, may clarify more details about their suffering. The researchers highly recommend establishing support groups and regular psychiatric checkups for physicians, especially those with more risk factors for depression and suicide.

## Conclusion

Suicide risk is higher among younger, single physicians of both sexes, as well as those with psychiatric or chronic medical disorders. More depressive symptoms are seen in physicians who have more extended hours or night shifts and who have fewer academic degrees. Almost all medical specialties face the same risk of depression and suicide. Longitudinal research is recommended for regular follow-up of suicidal thoughts and depressive symptoms.

## Data Availability

The datasets used during this study are available upon request from the corresponding author.

## Abbreviations

BDI-II: Beck Depression Scale
BSSI: Beck Scale for Suicidal Ideation
r: Correlation coefficient
SD: Standard deviation
SIDAS: Suicidal Ideation Attributes Scale
SPSS: Statistical Package for the Social Sciences
WHO: World Health Organization
